# Fasting blood glucose and cerebrospinal fluid Alzheimer’s biomarkers in non-diabetic cognitively normal elders: the CABLE study

**DOI:** 10.18632/aging.102921

**Published:** 2020-03-17

**Authors:** Ya-Nan Ou, Xue-Ning Shen, He-Ying Hu, Hao Hu, Zuo-Teng Wang, Wei Xu, Qiang Dong, Lan Tan, Jin-Tai Yu

**Affiliations:** 1 Department of Neurology, Qingdao Municipal Hospital, Qingdao University, Qingdao, China; 2 Department of Neurology and Institute of Neurology, Huashan Hospital, Shanghai Medical College, Fudan University, Shanghai, China

**Keywords:** fasting blood glucose, Alzheimer's disease, β-amyloid, cerebrospinal fluid, gender

## Abstract

It is unclear how blood glucose levels mediate the pathology of Alzheimer's disease (AD). This study aimed to investigate whether fasting blood glucose (FBG) levels are associated with cerebrospinal fluid (CSF) biomarkers preferentially affected by AD in non-diabetic cognitively normal elders. A total of 499 non-diabetic cognitively normal elders were from the Chinese Alzheimer’s Biomarker and LifestyLE (CABLE) study. We detected the associations of FBG with individual CSF measures using multiple linear regression models controlling for age, sex, educational level, and *apolipoprotein E* (*APOE)* ε4 genotype. Fasting blood glucose level was positively correlated with CSF Aβ_42_ level (β = 0.045, p = 0.010), CSF Aβ_42_/Aβ_40_ ratio (β = 0.005, p < 0.001), Aβ_42_/P-tau ratio (β = 0.282, p = 0.013), and Aβ_42_/T-tau ratio (β = 0.050, p = 0.040). Interaction analysis indicated that gender affected the correlations of FBG level with CSF Aβ_40_ (p < 0.001) and Aβ_42_/Aβ_40_ ratio (p < 0.001). This study raises additional questions about the role of blood glucose in the predisposition to AD and supports the possibility of targeting these processes in pre-symptomatic AD trials in non-diabetic elders.

## INTRODUCTION

Mounting evidence supports the notion that type 2 diabetes mellitus (T2DM) could increase the risk of Alzheimer’s disease (AD) [1–3] via multiple possible pathways, including cerebrovascular disease and neurodegeneration [2]. However, inconsistencies emerge in the underlying mechanisms concerning the neurodegenerative process. One study suggested that the neurodegenerative effects of T2DM might be driven by pathways that promote neuronal tau more than β-amyloid (Aβ) [4]. However, a previous cohort study among non-diabetic AD patients found that cerebrospinal fluid (CSF)/plasma glucose ratio was inversely related with CSF Aβ_42_ level rather than CSF tau level [5]. Moreover, one study indicated that Aβ_42_ in CSF was positively associated with T2DM status [6]. Furthermore, most previous studies that have examined associations between glucose metabolism and neuropathological outcomes have focused on diabetes itself [7], and far fewer studies have specifically examined fasting blood glucose (FBG), which is better in reflecting the level of blood glucose as well as its association with AD biomarkers than the diagnosis of DM. Elevated fasting serum glucose has been linked to decreased memory functioning in cognitively normal (CN) older adults and may be a risk factor for cognitive impairment or predisposition to AD [8]. Therefore, AD prevention research may benefit from the research on the association between elevated fasting blood glucose and AD biomarkers in non-diabetic cognitively normal individuals.

Our study aimed to explore the cross-sectional relationships between FBG and CSF biomarkers of neurodegeneration [CSF Aβ_42_, Aβ_40_, phosphorylated-tau (P-tau), and total-tau (T-tau)] usually implicated in the development of AD in non-diabetic cognitively normal elders gathered from Qingdao, China. We also tested the interactions of gender and *apolipoprotein E (APOE)* ε4 status with FBG levels related to AD biomarkers in CSF.

## RESULTS

### Basic characteristics of study population

Table 1 demonstrates the demographic and clinical characteristics of the whole study population. A total of 499 non-diabetic cognitively normal elders were included in our analysis, who were in their late adulthood (61.13 ± 10.62 years old) and had 9.77 ± 4.38 years of education, with a slight male predominance (57.8%), a small proportion of *APOE* ε4 allele positivity (15.4%), and a mean Mini-Mental State Examination (MMSE) score of 27.89 ± 2.12.

**Table 1:** Basic characteristics of participants included.

**Characteristics**	**Total population**
Age (years)	61.13 ± 10.62
Female (%)	211 (42.3%)
Educational level (years)	9.77 ± 4.38
*APOE* ε4 positive (%)	77 (15.4%)
FBG (mmol/L)	5.49 ± 1.05
MMSE score	27.89 ± 2.12
CSF biomarkers (pg/mL)	
Aβ_42_ (n = 453)	162.76 ± 101.59
Aβ_40_ (n = 496)	6344.83 ± 2916.52
P-tau (n = 499)	38.39 ± 10.45
T-tau (n = 482)	174.25 ± 86.87

Continuous variables are presented as mean ± SD, and categorical variables as number (percentage). Abbreviations: FBG, fasting blood glucose; *APOE* ε4, *apolipoprotein E* ε4; MMSE, mini-mental state examination; CSF, cerebrospinal fluid; Aβ, β-amyloid; P-tau, phosphorylated-tau; T-tau, total-tau.

### Correlations between FBG and CSF biomarkers of AD

**Figure 1:**
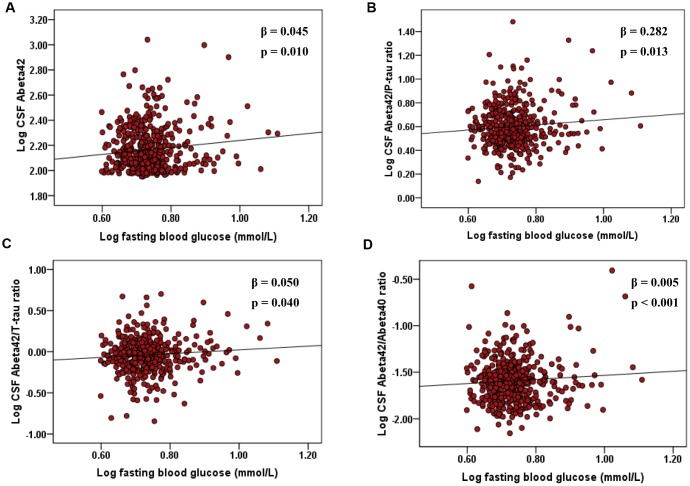
**Associations of elevated FBG with CSF Aβ_42_, Aβ_42_/P-tau ratio, and Aβ_42_/T-tau ratio, and Aβ_42_/Aβ_40_ ratio in non-diabetic cognitively normal elders.** The scatter plots depict the relations between FBG and (**A**) CSF Aβ_42_, (**B**) Aβ_42_/P-tau ratio, and (**C**) Aβ_42_/T-tau ratio, and (**D**) Aβ_42_/Aβ_40_ ratio. All models were adjusted for age, sex, educational level, and *APOE* ε4 status. Abbreviations: FBG, fasting blood glucose; CSF, cerebrospinal fluid; Aβ, β-amyloid; T-tau, total-tau; phosphorylated-tau, P-tau; *APOE* ε4, *apolipoprotein E* ε4.

The results on associations of fasting blood glucose with CSF biomarkers of AD in the non-diabetic cognitively normal elders were shown in Table 2 and Figure 1. When adjusting for age, sex, educational level, and *APOE* ε4 status, higher FBG levels were associated with elevated CSF Aβ_42_ levels (n = 453; β = 0.045, p = 0.010), CSF Aβ_42_/Aβ_40_ ratio (n = 444; β = 0.005, p < 0.001), Aβ_42_/P-tau ratio (n = 434; β = 0.282, p = 0.013), and Aβ_42_/T-tau ratio (n = 431; β = 0.050, p = 0.040), but not associated with CSF Aβ_40_ (n = 496), P-tau (n = 499), or T-tau (n = 482) levels in the non-diabetic CN elders (Table 2). Interaction analysis indicated that gender affected the associations of elevated FBG levels with higher CSF Aβ_40_ level and Aβ_42_/Aβ_40_ ratio (all p < 0.001; Table 2). Specifically, subsequent stratified analyses showed that among women, FBG was positively correlated with CSF Aβ_40_ level (n = 208; β = 0.070, p = 0.010), but negatively related among the males (n = 288; β = -0.072, p = 0.017). However, only among the males, FBG was significantly associated with higher CSF Aβ_42_/Aβ_40_ ratio (n = 258; β = 0.010, p < 0.001; Table 3). *APOE* ε4 genotype was not a modifier.

**Table 2:** Associations between elevated FBG and CSF biomarkers non-diabetic CN elders.

**β (p)**	**FBG**	**Interactions (p value)**	
		**Gender**	***APOE* ε4**
Aβ_42_	**0.045 (0.010)**	0.078	0.600
Aβ_40_	-0.002 (0.925)	**<0.001**	0.858
P-tau	0.013 (0.192)	0.597	0.291
T-tau	0.010 (0.521)	0.178	0.792
Aβ_42_/Aβ_40_	**0.005 (<0.001)**	**<0.001**	0.079
Aβ_42_/P-tau	**0.282 (0.013)**	0.287	0.591
Aβ_42_/T-tau	**0.050 (0.040)**	0.655	0.581

Bold figures represent significant correlations. Abbreviations: FBG, fasting blood glucose; CSF, cerebrospinal fluid; Aβ, β-amyloid; P-tau, phosphorylated-tau; T-tau, total-tau; *APOE* ε4, *apolipoprotein E* ε4.

**Table 3:** Associations between elevated FBG and CSF biomarkers stratified by gender.

**β (p)**	**Female**	**Male**
Aβ_40_	**0.070 (0.010)**	**-0.072 (0.017)**
Aβ_42_/Aβ_40_	<0.001 (0.857)	**0.010 (<0.001)**

Bold figures represent significant correlations. Abbreviations: FBG, fasting blood glucose; CSF, cerebrospinal fluid; Aβ, β-amyloid.

## DISCUSSION

This is a population-based cross-sectional study to explore the correlations between FBG and various AD-related CSF biomarkers in non-diabetic cognitively normal elders. Positive correlations of FBG levels with CSF Aβ_42_ level, Aβ_42_/Aβ_40_ ratio, Aβ_42_/P-tau ratio and Aβ_42_/T-tau ratio were found. However, we did not find evidence of significant relationships between FBG and CSF tau levels. Interaction analysis suggested that the associations of FBG with CSF biomarkers may be mediated by gender.

In the present study, we discovered that FBG levels were positively associated with CSF Aβ_42_ levels in cognitively normal elders without T2DM. No evidence was found for significant relationships between FBG and CSF tau (neither T-tau nor P-tau) levels. These results are consistent with a previous cohort study among non-diabetic AD patients which found that CSF/plasma glucose ratio was inversely related with CSF Aβ_42_ level rather than CSF tau level [5]. The early plaque accumulation can be theoretically due to Aβ_42_ overproduction, as seen in familial AD [9], or defective clearance [10] during the period without cognitive impairment. Preclinical studies in mouse models of cerebral amyloidosis suggest that systemic hyperglycemia increases Aβ levels within the hippocampal interstitial fluid (ISF); an effect that is exacerbated in aged AD mice with marked Aβ plaque pathology during the hyperglycemia challenge [11, 12]. Moreover, mouse plasma glucose and ISF Aβ are highly correlated, and elevated glucose levels drive Aβ production in the hippocampus in an activity dependent manner [12]. As for the ratios, several studies have reported that the CSF Aβ_42_/Aβ_40_ ratio could outperform CSF Aβ_42_ as a more accurate marker of brain amyloidosis, since it normalizes the CSF Aβ_42_ levels according to the total production of Aβ in the brain [13, 14]. The positive association between elevated FBG and CSF Aβ_42_/Aβ_40_ ratio was discovered, suggesting that FBG may increase the incidence of Aβ aggregation (maybe through increased amyloidogenic APP processing) [10]. Moreover, the combination of CSF Aβ_42_/P-tau ratio and Aβ_42_/T-tau ratio has been proposed to provide more accurate risk assessments for the development of AD [15–17]. These ratios reflect two aspects of AD pathology, i.e., plaques (Aβ_42_), and neurodegeneration (tau) [18]. Higher FBG levels were also found to be associated with greater CSF Aβ_42_/P-tau ratio and Aβ_42_/T-tau ratios.

Actually, most previous studies that have examined associations between glucose metabolism and neuropathological outcomes of AD have focused on diabetes, finding that T2DM poses a great known risk (2- to 4- fold) for developing AD. Both of the two diseases share common pathologies albeit they have been identified in the periphery with T2DM and in the brain with AD. These include an increase in inflammation, oxidative stress, adiponectin deficiency, plasma cholinesterase activity, vascular damage, dysregulation of glucose and insulin signaling [19–21]. Although it has been suggested that T2DM pathologies can promote early neurodegenerative processes, the mechanisms by which diabetes modifies AD and the mechanisms underlying diabetes-associated peripheral neuropathy remain unclear. However, notably, in the non-diabetic population we are focusing on, the overall blood glucose level is not high enough to have these diabetes-like characteristic pathological changes mentioned above. In the present study, the results indicated that hypoglycemia was a risk factor for AD pathology, and hyperglycemia (within an appropriate level) played a protective role in AD-related biological changes, which is consistent with previous reports. Several studies suggest the possibility that repeated and/or severe hypoglycemia could lead to an elevated risk of dementia [19–21]. As far as we know, we are the first to detect the correlations of FBG level and AD core biomarkers in non-diabetic cognitively normal elders. There are several potential reasons that it could lead to an increased risk for dementia. Firstly, hypoglycemia can lead to neuronal cell death, which may be of particular concern in older patients with limited neuronal plasticity. Furthermore, hypoglycemia increases platelet aggregation and fibrinogen formation and could conceivably lead to microvascular events. Finally, hypoglycemia can damage receptors in regions of the brain critical for learning and memory [19]. However, the exact pathological mechanism is poorly understood and demands future investigations.

These positive relationships between FBG and CSF biomarkers were demonstrated in the CN elders. As it is nowadays contextualized, AD has a long asymptomatic period in which there is a silent accumulation and progression of pathology and brain structural changes. Symptoms appear when compensatory mechanisms have been overcome, initially as mild cognitive impairment (MCI) and ultimately as dementia [22, 23]. This result may give us a hint that elevated blood glucose may be mainly associated with higher CSF biomarkers in the stage without cognitive impairment. This study raises additional questions about the role of blood glucose in the predisposition to AD and supports the possibility of targeting these processes in pre-symptomatic AD trials in non-diabetic elders. Furthermore, interaction analysis indicated that gender affected the correlations of FBG levels with CSF Aβ_40_ and Aβ_42_/Aβ_40_ levels. Specifically, FBG was positively associated with CSF Aβ_40_ levels among the females, but negatively related among the males. Only among males, FBG was significantly associated with higher CSF Aβ_42_/Aβ_40_ ratio. This is the first study to detect the sex differences in the relationships between blood glucose and AD biomarkers in CSF. The exact mechanism remains unclear. Further studies are warranted to clarify the essence of gender differences and the underlying mechanisms.

Some limitations should be acknowledged. Firstly, the present study is limited by its cross-sectional design, therefore limiting inferences of causality. Further longitudinal analyses will assist in establishing whether the associations support causality. Next, because of the smaller proportion of people who were *APOE* ε4 positive, we were unable to link the impact of the *APOE* ε4 risk allele on the association of FBG with levels of AD biomarkers. In addition, our measures of glucose. metabolism are limited to peripheral, rather than cerebral measures. However, this adds to the current literature by indicating that peripheral hyperglycemia, which is very easy to measure with noninvasive measures, is related to AD-related CSF biomarkers. Lastly, the level of fasting blood glucose can be influenced by many factors, including time related to meal, type of meal, and exercise pattern. Future studies regarding the correlations of plasma level of glycosylated hemoglobin (HbA1C), a more stable value related blood glucose status, and AD-related core biomarkers are needed.

Despite these limitations, we are the first to investigate the relationship between FBG and Alzheimer’s CSF biomarkers in the non-diabetic CN elders. Our finding is especially important when taking into consideration the public health impacts of AD coupled with epidemiological data showing that rates of this disease states are expected to dramatically increase. Our study advanced the association between AD and blood glucose level to the stage where no cognitive impairment occurred and diabetes did not develop. Among cognitively normal elders without diabetes, maintaining appropriate levels of hyperglycemia demonstrated a protective effect on AD pathology, whereas low glycemic level were adverse. Hypoglycemia could cause the pathogenesis of AD by the way described above. Older individuals are thought to have less brain reserve or brain plasticity [24, 25], and therefore may be unable to recover from neurological insult. So, it is important for the elderly to maintain an appropriate level of blood glucose. However, given that this present study is cross-sectional, and thus it complements more time-consuming prospective cohort studies in the assessment of FBG and AD risk. It is also important to note that even the notion that diabetes is positively correlated with the high risk of AD is now generally accepted, the relationship between FBG and the risk of AD pathology, in the population with obvious cognitive impairment, and in the population with diabetes, needs to be further explored.

In summary, this study provides additional evidence for the relationship between higher fasting blood glucose levels and the predisposition to AD. It demonstrated that, in non-diabetic cognitively normal elders, higher FBG levels were associated with elevated CSF Aβ_42_ level, Aβ_42_/Aβ_40_ ratio, Aβ_42_/P-tau ratio, and Aβ_42_/T-tau ratio. Gender significantly modified the association between elevated blood glucose and AD pathology. However, the underlying pathological mechanisms warrant further investigation in the future.

## MATERIALS AND METHODS

### Participants

Non-diabetic cognitively normal northern Han Chinese participants were from the Chinese Alzheimer’s Biomarker and LifestyLE (CABLE) study. CABLE is an ongoing large-scale study majorly focused on risk factors and biomarkers for AD in Chinese Han population since 2017, aiming to determine the genetic and environmental modifiers of AD biomarkers and their utility in early diagnosis. Individuals were recruited at Qingdao Municipal Hospital, Shandong Province, China. All enrolled participants were Han Chinese aged between 40 to 90 years. The exclusion criteria include: (1) central nervous system infection, head trauma, epilepsy, multiple sclerosis or other major neurological disorders; (2) major psychological disorders; (3) severe systemic diseases (e.g., malignant tumors); (4) family history of genetic diseases. All participants gave written informed consent to participate in this study, which was approved by the Institutional Review Board of Qingdao Municipal Hospital and conducted in accordance with the principles of the Declaration of Helsinki. This study only included those without T2DM diagnosis.

At study entry, detailed clinical assessment was performed including an interview with the care-giver and an extensive neurological examination. Standardized questionnaires were then evaluated. The China-Modified MMSE (CM-MMSE) and Montreal Cognitive Assessment (MoCA) scales were used to examine global cognition. The basic Activities of Daily Living score (ADL) was used to assess basic living ability; the Geriatric Depression Scale (GDS), Hamilton Rating Scale for Depression (HAMD) and Hamilton Rating Scale for Anxiety (HAMA) for behavioral or psychological symptoms. The GDS and HAMD scores of participants in CABLE database were less than seven. Moreover, vascular factors were assessed by Hachinski Inchemic Score (HIS). The HIS scores of participants in our study were less than four. Finally, this current study included 499 non-diabetic cognitively normal individuals with available data on CSF measures, FBG levels, and *APOE* ε4 genotypes.

### CSF biomarkers and FBG measurements

CSF sample collection and management was in accordance with the international consensus on standardization of CSF research [26]. CSF was collected via a lumbar puncture in the L3/L4 or L4/L5 intervertebral space, using a 25-gauge needle. In the case of traumatic lumbar puncture, the first 1-2 mL of CSF was discarded and sample was collected after transparency. CSF sample was sent to the laboratory at room temperature within 2 hours and centrifuged at room temperature for 10 min. Following centrifugation (10 min, 1,800g, 4°C), CSF was stored in aliquots at -80°C until further analysis. To avoid repeated freeze-thaw cycles, only one tube of CSF sample was take out in each analysis (the maximal number of freeze-thaw cycles is limited to two, preferably one). CSF Aβ_42_, Aβ_40_, T-tau, and P-tau levels were measured by enzyme-linked immunosorbent assays (ELISAs) using INNOTEST (Fujirebio Europe N.V.). All CSF samples were distributed randomly across plates and measured in duplicate. All the antibodies and plates were from a single lot in order to exclude variability between batches. Experiments were performed by experienced laboratory technicians blinded to clinical diagnosis and other clinical information. The mean intra-batch coefficient of variation (CV) was 4.86 ± 4.37% for Aβ_42_, 3.63 ± 2.84% for Aβ_40_, 2.34 ± 2.30% for P-tau, and 4.59 ± 3.49% for T-tau. The mean inter-batch CV was 9.60% for Aβ_42_, 8.12% for Aβ_40_, 11.04% for P-tau, and 12.15% for T-tau. CSF biomarker levels were corrected by inter-batch variation and corrected values were used for analyses.

A laboratory blood specimen to assess a patient's fasting blood level was obtained after the patient has abstained from eating for a minimum of 8 hours. Fasting blood glucose levels were measured by glucose hexokinase (HK) method using Glucose Reagent (Ningbo Ruiyuan Biotechnology Co., Ltd, China).

### Assessment of covariates

DNA extracted from 10 mL ethylene diamine tetraacetic acid (EDTA) blood samples using the QIAamp DNA Blood kit (Qiagen, Hilden, Germany), was used for *APOE* ε4 genotyping by a Multiplex SNaP shot. Participants were classified as *APOE* ε4 non-carriers (participants with no copies of the *APOE* ε4 gene), and *APOE* ε4 carriers (individuals with at least one copy of the *APOE* ε4 gene). General demographic data, including age, sex, and educational level, were collected via a structured questionnaire.

### Statistical analysis

CSF values outlying the mean ± 3 SD were regarded as extremes and then excluded when analyzed. Continuous variables are presented as mean ± SD, and categorical variables as number (percentage). The associations of continuous FBG increment with individual CSF measures were analyzed using multiple linear regression models adjusting for age, sex, educational level, and *APOE* ε4 genotype. All CSF variables in linear regression models were log-transformed to normalize the distributions and to facilitate comparisons between modalities. Models with interaction terms were performed in order to examine the modifying effect of gender and *APOE* ε4 genotype on the relationship between blood glucose level and CSF biomarkers. In case of significant interactions, stratified analyses were performed. All tests were 2-sided and statistical significance was set at p < 0.05. Analyses were performed using IBM SPSS Statistics 23.
